# Graphene Oxide-Coated Metal–Insulator–Metal SERS Substrates for Trace Melamine Detection

**DOI:** 10.3390/nano12071202

**Published:** 2022-04-03

**Authors:** Zhenming Wang, Jianxun Liu, Jiawei Wang, Zongjun Ma, Delai Kong, Shouzhen Jiang, Dan Luo, Yan Jun Liu

**Affiliations:** 1 Department of Electrical and Electronic Engineering, Southern University of Science and Technology, Shenzhen 518055, China; 12150025@mail.sustech.edu.cn (Z.W.); 11930844@mail.sustech.edu.cn (J.W.); 12031132@mail.sustech.edu.cn (Z.M.); kongdl@mail.sustech.edu.cn (D.K.); luod@sustech.edu.cn (D.L.); 2 Provincial Key Laboratory of Optics and Photonic Device, School of Physics and Electronics, Shandong Normal University, Jinan 250014, China; jiang_sz@126.com

**Keywords:** SERS, self-assembly, metasurface, surface plasmon, hot spot, sensitivity

## Abstract

Surface-enhanced Raman spectroscopy (SERS) has long been an ultrasensitive technique for trace molecule detection. However, the development of a sensitive, stable, and reproducible SERS substrate is still a challenge for practical applications. Here, we demonstrate a cost-effective, centimeter-sized, and highly reproducible SERS substrate using the nanosphere lithography technique. It consists of a hexagonally packed Ag metasurface on a SiO_2_/Au/Si substrate. A seconds-lasting etching process of a self-assembled nanosphere mask manipulates the geometry of the deposited Ag metasurface on the SiO_2_/Au/Si substrate, which attains the wavelength matching between the optical absorbance of the Ag/SiO_2_/Au/Si substrate and the excitation laser wavelength as well as the enhancement of Raman signals. By spin-coating a thin layer of graphene oxide on the substrate, a SERS performance with 1.1 × 10^5^ analytical enhancement factor and a limit of detection of 10^−9^ M for melamine is achieved. Experimental results reveal that our proposed strategy could provide a promising platform for SERS-based rapid trace detection in food safety control and environmental monitoring.

## 1. Introduction

In recent years, the surface-enhanced Raman spectroscopy (SERS) technique for the detection of dangerous substances in small volumes and extremely low concentrations has attracted extensive interest [1,2,3,4]. As known, SERS can provide single-molecular sensitivity [5,6,7,8,9,10], label-free detection [11,12,13], and high-throughput screening of a huge number of samples [14,15], which is well suited for on-site trace detection and identification [16,17,18]. Among numerous theoretical and experimental reports on SERS substrates [16,17,18,19,20,21,22,23], there are three main strategies to enhance the Raman signal intensity and decrease the detection limit, including enhancing the absorption of incident light [19,20,21,22], amplifying the far-field radiation of Raman scattering signals [23,24], and increasing the attachment of the targeted substances [25,26]. A universal approach to enhancing absorption of the excitation laser and Raman signals is to construct a substrate composed of dense metal nanostructures [27,28,29]. These nanostructures have not only large optical absorption cross sections but also numerous “hot spots” produced by the localized surface plasmon resonance (LSPR) to enhance the far-field radiation. Large Raman signal enhancement has been achieved in some SERS substrates with randomly dispersed metal nanostructures [21,30]. However, how to increase the absorption of the excitation laser has yet been received enough attention. A good way to achieve a SERS substrate with specific wavelength absorption is to artificially design plasmonic metasurfaces [22,29,31,32], which are a periodic arrangement of subwavelength meta-atoms with special shapes, such as nanodisks, nanopillars, nanogrids, etc. [33,34,35,36,37,38].

Among numerous kinds of plasmonic nanostructures, nanobowties have been demonstrated to achieve relatively large Raman signal enhancement [34]. In addition, the choice of metal materials will also affect the Raman enhancement. For the case of 532 nm excitation laser, silver nanostructures have a smaller photothermal effect than gold ones, leading to a larger enhancement factor [35,38]. Besides silver, other materials such as aluminum, lead, and platinum are also good choices, but silver could be the best choice with a much easier fabrication process [39,40,41,42]. A detection limit down to 10^−16^ M for Rhodamine 6G has been reported by silver metasurfaces formatted with nanobowties [35]. Additionally, it is worth noting that the reduced photothermal effect could help avoid damage to the analytes during the measurement process [43]. 

As a well-known poisoning additive, melamine is sometimes illegally adulterated with food products in order to increase the apparent protein content. Ingestion of melamine may cause reproductive damage, kidney stones/failures, and bladder cancer. In adulterated milk products, melamine concentrations could be as high as ~3300 ppm, far exceeding the safety limit of 1 ppm (~7.9 × 10^−6^ M), hence posing an extreme danger to consumers. Therefore, rapid and unambiguous detection of trace amounts of melamine in food products is of paramount importance. To counteract the adverse effects of melamine, various analytical techniques based on SERS for detecting it in dairy and food products have been developed [44,45,46,47]. For example, in 2016, Dong et al. [44] used polythymine aptamer-modified Au nanoparticles to decrease the limit of detection down to 8 × 10^−12^ M for melamine with the SERS effect. Li and co-workers [45] synthesized a monolayer film with Ag nanoparticles to realize a SERS effect on melamine with a limit of detection near 3 × 10^−13^ M. Such SERS substrates are characterized by the formation of a sufficient number of hot spots near or between nanoparticles to achieve the strong SERS effect. Huang and co-workers [46] demonstrated the low limit of detection of 1×10^−16^ M by using Ag nanoparticles decorated with Zinc oxide/Silicon heterostructured nanomace arrays as a SERS substrate. The large-area detection for extremely low concentration is realized; however, the SERS substrate fabrication process is very complicated and time-consuming. Kundan et al. [47] fabricated an Au nanorod array with a focused ion beam (FIB) and achieved the detection limit of 1 × 10^−12^ M for melamine. Although the low limit of detection for melamine is also achieved in a large area, the preparation processes are greatly dependent on precision nanofabrication techniques, such as electron beam lithography (EBL) and FIB, leading to complicated and costly fabrication. Therefore, it is still in high demand to develop large-area, uniform SERS substrate with a simple and cost-effective fabrication technique. More importantly, there is no artificially designed structure to purposely investigate the effect of the characteristic absorption of excitation light on SERS signal enhancement.

Self-assembly-based nanosphere lithography (NSL) is a simple, cost-effective approach compared with EBL and FIB. Nanospheres as tiny as 200 nm can be used for monolayer self-assembly that can be further used as a templated mask [48]. Dai et al. fabricated Au triangular nanoarrays covered by Au nanoparticles by NSL to achieve the detection limit of 1 × 10^−7^ M for melamine [49]. In our previous reports, we have devised a slightly modified self-assembly process to achieve a large-area, high-quality polystyrene (PS) nanosphere monolayer [50,51]. In this work, we fabricate a hexagonally packed Ag metasurface on SiO_2_-Au-coated silicon (Ag/SiO_2_/Au/Si) substrate based on the NSL technique, forming a metal–insulator–metal (MIM) SERS substrate. By etching the PS nanosphere of the template mask with the inductively coupled plasma (ICP), strong plasmonic coupling at the wavelength of 532nm is achieved between the tips of nanotriangles in the metasurface and the Ag metasurface with strong absorption for the excitation laser wavelength of 532 nm is fabricated purposely. By introducing the MIM (Ag/SiO_2_/Au/) structure, the characteristic absorption for the excitation laser wavelength of 532 nm can be further enhanced. To additionally improve the SERS effect, a very thin layer of graphene oxide (GO) is spin coated on the Ag/SiO_2_/Au/Si structure to increase the number of target molecules adsorbed on the structure. An analytical enhancement factor (AEF) of 1.1 × 10^5^ and a detection limit down to 8 × 10^−9^ M for melamine are achieved in the GO/Ag/SiO_2_/Au/Si SERS substrate. The experiment results reveal that our proposed MIM substrates exhibit a highly sensitive and reproducible SERS activity. They also feature a simple, low-cost, and large-area fabrication process. These advantageous features make our proposed MIM SERS substrates promising for efficient and rapid detection in food safety control and environmental monitoring.

## 2. Materials and Methods

### 2.1. Materials Preparation

Melamine (99.0% purity), GO aqueous solution (0.15 mg/mL, 2–5 layers dispersion in water), and PS latex nanosphere solution in diameter of 400 nm (2.5 wt.% dispersion in water) were purchased from Shanghai Aladdin Bio-Chem Technology Co., Ltd. (Shanghai, China). The silicon substrates were purchased from HWOTECH (Shenzhen, China). Various molar concentrations of aqueous melamine solutions from 10^−9^ to 10^−2^ M were prepared for SERS experiments. 

### 2.2. SERS Substrates Preparation

Interventional studies involving animals or humans, and other studies that require ethical approval, must list the authority that provided approval and the corresponding ethical approval code. To prepare the templated masks, a cleaned Si substrate was treated for 15 min with a UV-O_3_ cleaner (BZS250GF-TC, HWOTECH, Shenzhen, China). A 100 nm Au layer and a 100 nm SiO_2_ layer were subsequently deposited on another cleaned Si substrate. The templated mask was then prepared with an improved self-assembly method that was described in detail in our previous report [50]. When the SiO_2_/Au/Si substrate was ready, the templated mask was finally transferred onto the SiO_2_/Au/Si substrate for the silver metasurface fabrication before the water was fully evaporated. Upon transferring the templated mask onto the SiO_2_/Au/Si substrate, the PS nanospheres were etched by O_2_ plasma via ICP (GSE200Plus, Northern Microelectronics, Gateshead, England) technique. A flow of 50 sccm O_2_ was used with ICP power 100 W and bias power 20 W to etch the nanospheres for 17, 19, 21, and 23 s, respectively, at a pressure of 8 mTorr. After etching, a 3 nm Cr layer was deposited onto the etched templated mask as an adhesion layer, and a 100 nm Ag layer was deposited subsequently by an electron-beam evaporator (EBE, TF500, British HHV, Crawley, England). During the deposition process, a vacuum of 5 × 10^−6^ Torr was maintained within the evaporator chamber. The evaporation rates were controlled to be ~0.1 Å/s for Cr and ~0.8 Å/s for Ag. After deposition, the Ag-coated templated masks were immersed in deionized water, and the PS nanospheres were removed using ultrasonic cleaning. The removing process was 1 min under an ultrasonic power of 80 W. To further enhance the sensitivity, a GO solution (0.15 mg/mL, Shanghai Aladdin Bio-Chem Technology Co., Ltd., Shanghai, China) was diluted 10 times, and then a 40 µL GO suspension was spin coated on the Ag metasurface at 2000 rpm for 2 min. Finally, a GO/Ag/SiO_2_/Au/Si substrate was ready for SERS testing.

### 2.3. Characterization and Measurement

A field emission scanning electron microscopy (FESEM, Merlin, Zeiss, Jena, Germany) was used to examine the surface morphologies of the Ag metasurface. A UV-Vis-NIR microspectrophotometer (CRAIC Technologies Inc., Altadena, California, United States) was used to investigate the optical properties of the fabricated MIM substrate. For the Raman spectra measurement, we used a 100× objective lens with a numerical aperture of 0.8 to focus the 532 nm laser beam onto the samples and collected the Raman signal with the spectrometer in a confocal Raman system (Alpha300, WITec, Ulm, Germany). The power of the excitation laser was 160 µW/µm^2^, and the acquisition time was 10 s for each spectrum. For the Raman mapping, we used a 100× objective lens to focus the excitation laser onto the GO/Ag/SiO_2_/Au/Si substrate with the melamine concentration of 10^−5^ M. The power of the laser beam was 160 µW/µm^2^, and the integrating time was 0.1 s for each point.

## 3. Results

**Figure 1:**
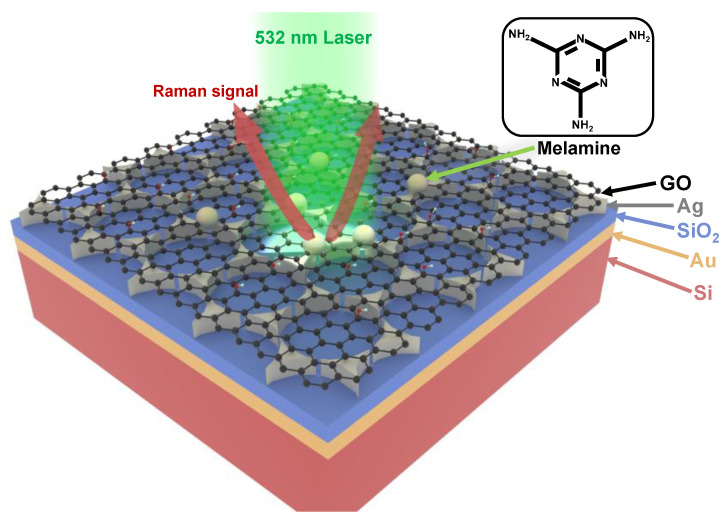
Schematic illustration of trace melamine measurement using the GO/Ag/SiO_2_/Au/Si SERS substrate. The gray spheres represent the melamine molecules. The right top illustrates the molecular structure of melamine.

Figure 1 shows the schematic for trace melamine measurement using the GO/Ag/SiO_2_/Au/Si SERS substrate. The MIM structure consists of a 100 nm Au layer, 100 nm SiO_2_ layer, and 100 nm Ag metasurface layer, respectively, from the bottom to the top. The coated GO layer has a thickness of ~2 nm. The inset in Figure 1 illustrates the molecular structure of melamine, whose Raman signals mainly come from two vibration modes [47]. One mode is that the three C atoms in the central ring are stretched out-of-plane to form a scissor oscillation, which drives the three amino groups to move together; and the other one is that the three N atoms of the central ring oscillate out-of-plane instead of C atoms, with which there is no oscillation for the three amino groups. Correspondingly, under the 532 nm laser excitation, two major peaks on the SERS spectrum can be clearly observed, which will be discussed later. 

**Figure 2:**
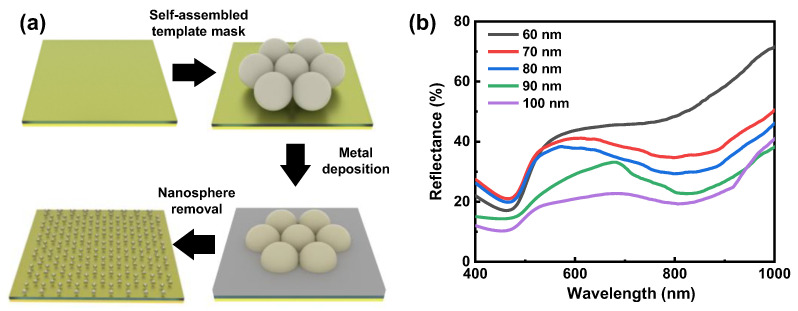
(**a**) Schematic fabrication processes for the Ag metasurfaces without ICP etching. (**b**) Measured reflection spectra with different insulator gaps.

In the MIM structure, the Au layer serves as a reflection mirror. To calculate the absorbance, we can directly use *A* = 1 − *R* since there is no light passing through the Au layer. In the beginning, we first optimized the insulator gap to achieve relatively strong absorption. Figure 2a shows the schematic fabrication processes for the Ag metasurfaces without ICP etching. With the fixed Ag metasurface, we measured the reflection spectra with different insulator (i.e., SiO_2_) gaps, as shown in Figure 2b. It can be seen from Figure 2b that as the SiO_2_ gap increases, the overall reflectance of the MIM structure decreases, indicating increased absorption. Given the excitation laser wavelength of 532 nm, we have chosen a 100 nm-thick SiO_2_ layer as the insulator gap.

In our design, the purpose is to achieve strong absorption of the excitation laser as well as the enhancement of Raman signals. Therefore, the Ag metasurfaces have to be deliberately designed and fabricated with plasmonic resonances, well suiting the laser wavelength and enhancing the Raman signals. Various strategies have been developed to modify the nanostructures based on the NSL technique [52,53,54,55,56,57,58,59,60]. However, the main strategy of these works mainly focuses on generating the hot spots, and the plasmonic couplings inside the metasurface have not been sufficiently investigated. The proposed strategy in our work is to slightly dwindle the nanosphere via ICP etching, producing the desired nanogaps between the nanotriangles to realize plasmon coupling and achieve strong absorption of excitation laser with the operating wavelength of 532 nm. The experimental results show that compared to non-etched template masks, the achieved MIM structures from etched template ones can provide much stronger optical absorption as well as deliberately tuned absorption peak matching the excitation laser wavelength [61,62,63].

**Figure 3:**
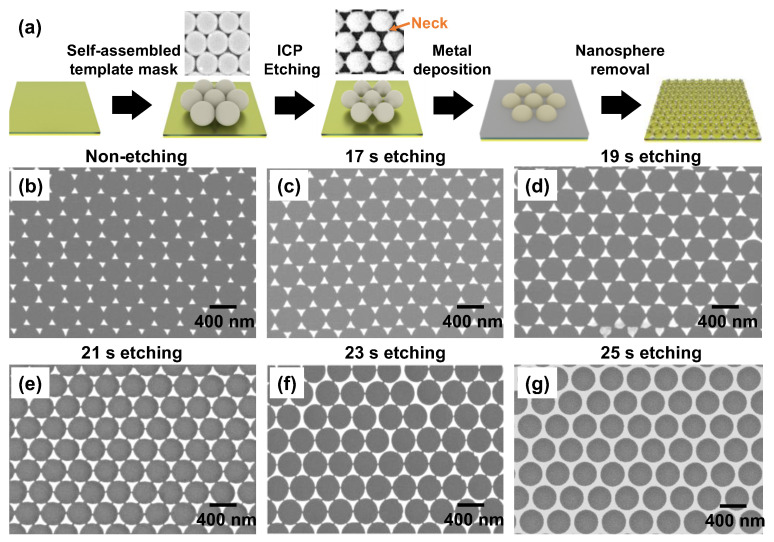
(**a**) Schematic fabrication processes for the Ag metasurfaces with ICP etching. Typical SEM images of the templated masks are comparatively illustrated before and after ICP etching. (**b**–**g**) SEM images of the fabricated Ag metasurfaces without and with ICP etching, respectively.

Figure 3a shows the schematic fabrication processes for the Ag metasurfaces with ICP etching. Figure 3b–g show the fabricated Ag metasurfaces without and with ICP etching, respectively. We can see from Figure 3a that for the non-etched templated mask, every two adjacent PS nanospheres will stick together, and all the nanospheres form a close-packed array (see the SEM image in Figure 3a). As a result, when depositing the Ag film, the entrance area is small, forming a metasurface with isolated nanotriangles in Figure 3b. The gap between the tips of the isolated nanotriangles is too far to form strong plasmonic couplings. While for the etched templated mask, we can reduce the size of nanospheres via ICP etching. With precise control, a thin bond can be formed between every two adjacent nanospheres, which is named as “neck”, as shown in the SEM image of Figure 3a. As the etching time increases, the size of the nanospheres further decreases, and the neck becomes longer and thinner. Therefore, we can adjust the size and the gap distance between the tips of the deposited Ag nanotriangles, as shown in Figure 3c–f. It is worth noting that for an etching time of 25 s, nanotriangles in the metasurface will connect to form a holey array, as shown in Figure 3g.

**Figure 4:**
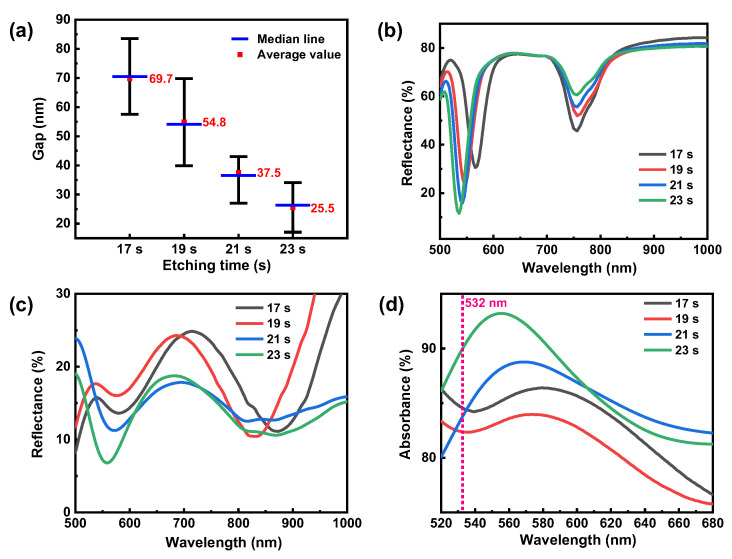
(**a**) Measured the gap distances between the adjacent nanotriangles at different etching times. (**b**,**c**) Simulated and measured reflection spectra of the MIM substrate with different gaps. (**d**) Measured absorbance spectra of the MIM substrate with different gaps.

To find the optimal etching time for the desired Ag metasurfaces, we measured the gaps between the adjacent nanotriangles when the templated masks were etched at different times from 17 to 23 s, as shown in Figure 4a. As the etching time increases, the gap decreases almost linearly from ~70 to 26 nm. Correspondingly, we have simulated and measured the reflection spectra of the MIM substrate with different gaps, as shown in Figure 4b,c. Overall, a similar trend can be observed from both simulation and experimental results. We can see that the reflection dip within the near-infrared range becomes shallower with the size increase in the nanotriangles, indicating a gradually weak resonance. In contrast, the reflection dip within the visible range becomes deeper, indicating a gradually strong resonance. At the etching time of 17 s, there is a resonance at a wavelength of ~581 nm from isolated nanotriangles. As the etching time increases, the resultant resonance blueshifts, as clearly shown from the absorbance spectra in Figure 4b,c. At the etching time of the 23 s, a deep dip with only ~7% reflectance at the wavelength of 555 nm can be clearly observed from the reflection spectrum. The measured absorbance spectra for MIM structures with Ag metasurfaces fabricated by different etching times are shown in Figure 4d. Therefore, one can tune the MIM structure’s absorption to match the excitation laser wavelength as much as possible. It is worth mentioning that for the fabricated MIM structures without ICP etching, there is only an LSPR centering at ~820 nm from the isolated nanotriangles, while for the Ag metasurface with ICP etching, a new resonance dip appears in the visible range, which is mainly attributed to nanogap-induced LSPR coupling.

**Figure 5:**
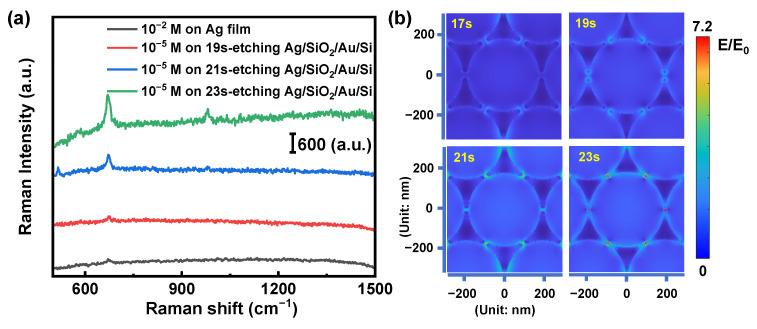
(**a**) Measured SERS spectra for the 10^−5^ M melamine on the Ag/SiO_2_/Au/Si substrates with different etching times of 19 s, 21 s, 23 s, and 10^−2^ M melamine on the Ag film substrate, respectively. (**b**) Electric field distributions on the nanostructured surface with different etching times. Scale bar: 100 nm.

To further confirm that the coupling came into being during the etching process, Raman spectra measurement for melamine was carried out to see the relationship between the degree of etching and the intensity of the Raman signal. Figure 5a shows the SERS signal of 10^−5^ M melamine on Ag/SiO_2_/Au/Si structures with different etching times from 19 s to 23 s, respectively. We can see that for the same melamine concentration, the SERS signal increases with the increase in etching time. The Ag/SiO_2_/Au/Si structures with the 23 s etching time demonstrate the highest Raman intensity. As a control experiment, the Raman signal of melamine was also collected for the bare Ag film. From the experimental results, we can confirm that the SERS performance is highly related to the structural geometry in addition to the Ag itself. To further clarify the change of the coupling between the dips of triangles during etching, the electric field distributions of the nanostructure with different etching times are shown in Figure 5b. In our simulation, the excitation laser wavelength is 532 nm, and the gap distances are retrieved from Figure 4a. Figure 5b clears shows that with the etching time increasing from 19 s to 23 s, the plasmonic coupling between gaps of the triangles gets stronger. Both the experimental and simulated results confirm that the plasmonic resonances and couplings of the metasurfaces play a key role in the SERS signal enhancement.

As known, GO can help adsorb much more targeted molecules and hence chemically enhance the SERS activity [64,65,66]. Therefore, GO-coated plasmonic SERS substrates could have much higher sensitivity and lower detection limits due to a combination of chemical enhancement of GO and the number of hot spots of plasmonic nanostructures. Various methods have been proposed to form thin GO films on structures, such as dip coating, spin coating, and electric-field-assisted coating [67,68,69,70]. For electric-field-assisted coating, the Ag oxidation could be accelerated with the exposure to plasma or chemical solution with electric charges. Both spin coating and dip coating are facile, efficient approaches. However, spin coating is a favorite to achieve the large-area, uniform GO films.

**Figure 6:**
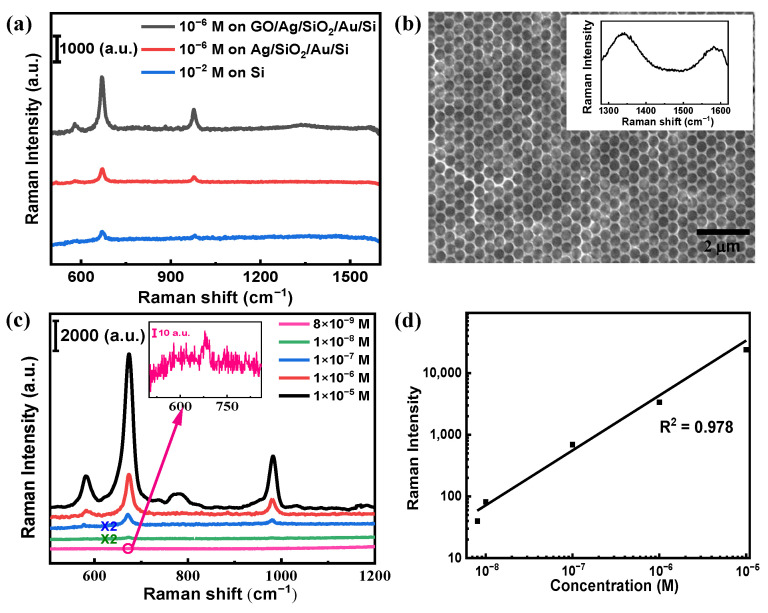
(**a**) Measured SERS spectra with 10^−6^ M melamine on the GO/Ag/SiO_2_/Au/Si substrate, the Ag/SiO_2_/Au substrate, and 10^−2^ M melamine on the Si substrate, respectively. (**b**) SEM image of GO-coated Ag metasurface. The inset shows the Raman spectrum of GO on the metasurface. (**c**) Measured SERS spectra for melamine with different concentrations from 1 × 10^−5^ M to 8 × 10^−9^ M on the GO/Ag/SiO_2_/Au/Si substrate. The inset illustrates the Raman peak of trace melamine with a concentration of 8 × 10^−9^ M at 671 cm^−1^. (**d**) Raman intensity of melamine at 671 cm^−1^ as a function of different concentrations in log scale.

We, therefore, further investigated the SERS performance of the GO-coated MIM substrate. In the Raman spectrum of melamine under excitation of 532 nm laser, there are two major peaks located at 671 cm^−1^ and 981 cm^−1^, as shown in Figure 6a, which correspond to the ring breathing vibration and the CNC + NCN bending vibration, respectively [71]. For the peak at 671 cm^−1^, the corresponding wavelength of the excited Raman signals is 552 nm, which is very close to the coupled resonance wavelength of 555 nm from the Ag/SiO_2_/Au/Si substrate with the etching time of 23 s. Therefore, we can enhance the Raman signals via both the Purcell effect [51,72,73,74] and the increased absorption efficiency of the excitation light. Figure 6b shows the SEM image of the GO-coated MIM substrate. The inset in Figure 6b illustrates the measured Raman spectrum with two clearly observed peaks at 1356 cm^−1^ and 1601 cm^−1^, corresponding to the D and G bands of GO, respectively [65]. Figure 6a shows the SERS signal of 10^−6^ M melamine on the GO/Ag/SiO_2_/Au substrate, the Ag/SiO_2_/Au substrate, and the Raman signal of 10^−2^ M melamine on the Si substrate, respectively. We can see that with the GO-coating, the Raman signals can be greatly enhanced. The analytical enhancement factor (AEF) [70] is calculated using the equation below to further quantify the enhanced distribution of the SERS substrate:

AEF = (I_SERS_ × C_REF_)/(I_REF_ × C_SERS_) (1)

where I_SERS_ and I_REF_ represent the peak intensity of the Raman signal from the GO/Ag/SiO_2_/Au/Si substrate and the Raman signal obtained from the Si substrate, respectively. C_SERS_ and C_REF_ are the concentrations of melamine solutions dropped on the SERS and reference substrates, respectively. The peak at 671 cm^−1^ is chosen to calculate the AEF. 

Herein, the measured I_SERS_ and C_SERS_ are 2450 and 10^−6^ M for melamine on the SERS substrate, and the measured I_REF_ and C_REF_ are 227 and 10^−2^ M for the reference substrate. Thus, the calculated AEF is 1.1 × 10^5^, indicating a high level of SERS performance of the GO-coated MIM substrate. We explored the sensitivity of the optimal SERS substrates further. Figure 6c shows the measured SERS spectra with different melamine concentrations. We can achieve the limit of detection (LOD) as low as 8 × 10^−9^ M for the melamine. Figure 6d shows the intensity of the typical Raman peak at 671 cm^−1^ as a function of melamine concentration on a log scale, demonstrating excellent linear dependency with a correlation coefficient (R^2^) of 0.978.

**Figure 7:**
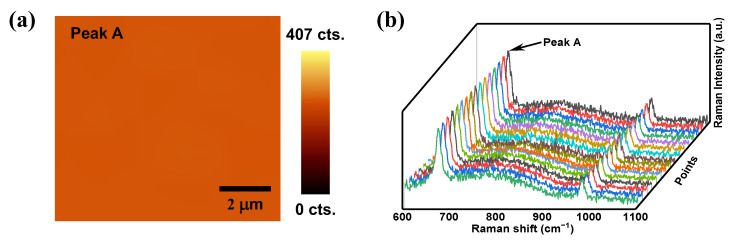
(**a**) Raman mapping of 10^−5^ M melamine with the collected signal of peak A at 671 cm^−1^. (**b**) Collected Raman signals of 10^−5^ M melamine at 16 randomly selected positions within the Raman mapping area.

For SERS-based molecular detection, in addition to the high sensitivity, uniformity is another important factor for practical applications. We carried out a 10 × 10 µm^2^ Raman mapping for uniformity evaluation of the modified SERS substrate surface. Figure 7a,b shows the Raman mapping result and the measured SERS spectra of melamine at 16 random positions within the Raman mapping area. It is obvious that the obtained profiles of spectra are almost identical, with neither a significant shift of the characteristic Raman peaks nor the obvious change of the peak intensity, indicating excellent uniformity of the fabricated SERS substrate.

## 4. Conclusions

In summary, we have demonstrated a facile, cost-effective strategy to achieve highly sensitive and reproducible SERS substrates using a modified NSL technique. Through precise control of nanosphere etching time, we can tune the MIM structure’s absorption to match the excitation laser wavelength as much as possible so as to enhance the light-MIM structure interactions. More importantly, we can precisely control the gap of the Ag nanotriangles to strong plasmonic couplings to further enhance the Raman signals. We have achieved a high-performance SERS activity for melamine with the AEF of 1.1×10^5^ and LOD of 10^−9^ M within a large area under the 532 nm laser excitation. Our proposed strategy could enable highly sensitive and reproducible SERS substrates with a simple, low-cost, and large-area fabrication process, hence providing a promising platform for SERS-based rapid trace detection in food safety control and environmental monitoring.

## Data Availability

The data presented in this study are available on request from the corresponding author.
